# Supplementary material for: Long non-coding RNA RAMS11 promotes metastatic colorectal cancer progression
Source: Nat Commun. 2020 May 1;11:2156. doi: 10.1038/s41467-020-15547-8 (PMC7195452; doi:10.1038/s41467-020-15547-8)
Supplement: Supplementary file 1 — Supplementary Information [file 41467_2020_15547_MOESM1_ESM.pdf]

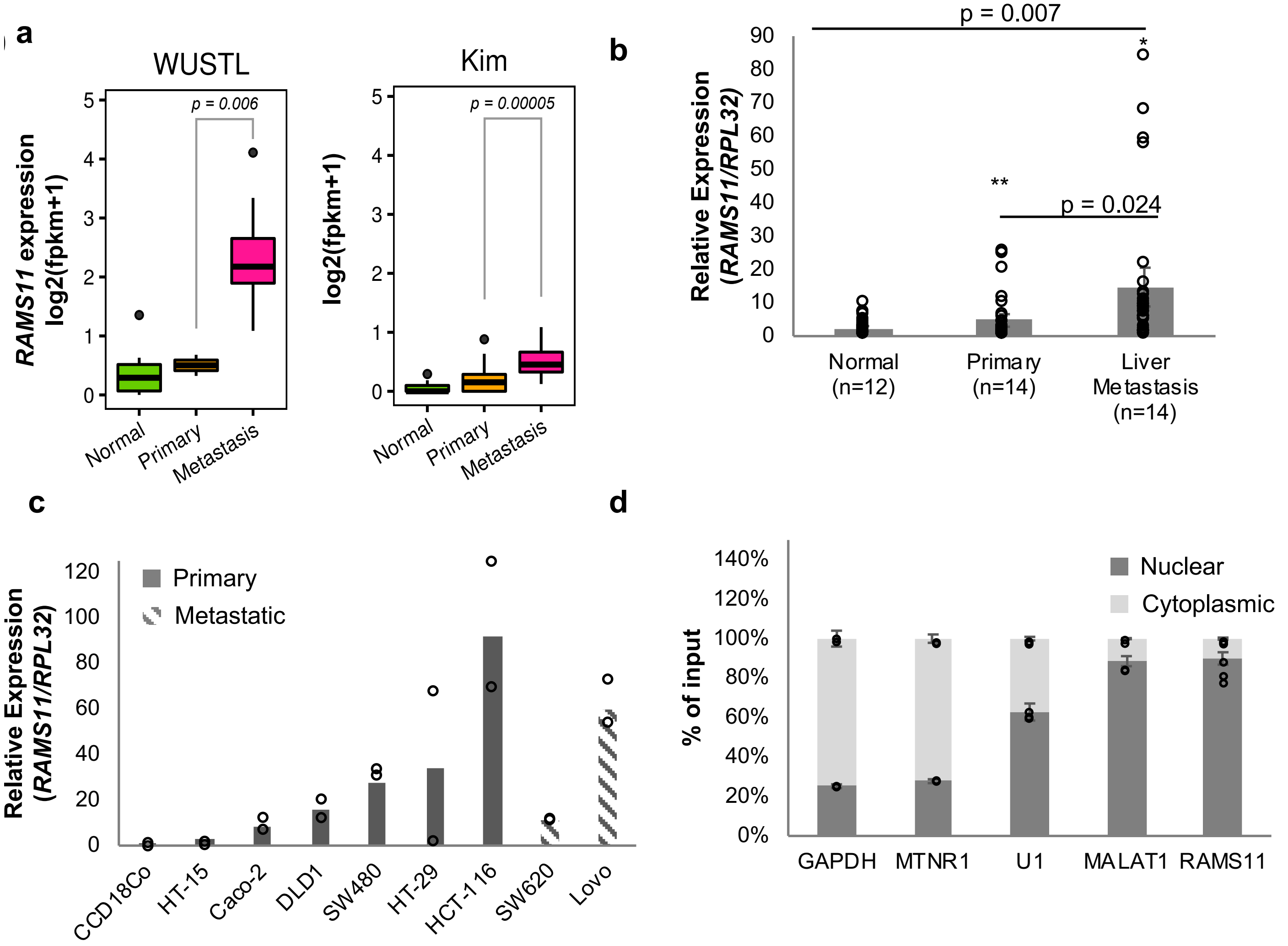

Supplementary Figure. 1: *RAMS11* expression in cell lines and patient tissues. (a) Expression of *RAMS11* in WUSTL and Kim cohorts. Shown are boxplots with box representing the interquartile range (IQR, 25th to 75th percentile) centered by median, whisker lines limited by 1.5xIQR away from the box, and dots representing outliers. WUSTL Normal n = 10, Primary n = 2, Metastasis n = 14, Kim Normal n = 18, Primary n = 18, Metastasis n = 18 **(b)** qPCR validation of matched normal, primary, and metastatic patient samples showing increased *RAMS11* expression in metastatic samples. Normal n = 12, Primary n = 14, Liver Metastasis n = 14. Data shown as mean ± SEM. **(c)** Expression of *RAMS11* in colon cancer cell line panel. Experiment repeated two times. **(d)** *RAMS11* is localized in the nucleus as shown by nuclear and cytoplasmic extraction. *GAPDH* and *MTNR1* genes were used as positive known cytoplasmic control genes, and *U1* snoRNA and *MALAT1* lncRNA were used as positive known nuclear localized control genes. Data is presented as mean values ± s.d. Experiment repeated three times. All data is analyzed by two-tailed paired *t*-test. *p < 0.05, ** p < 0.005

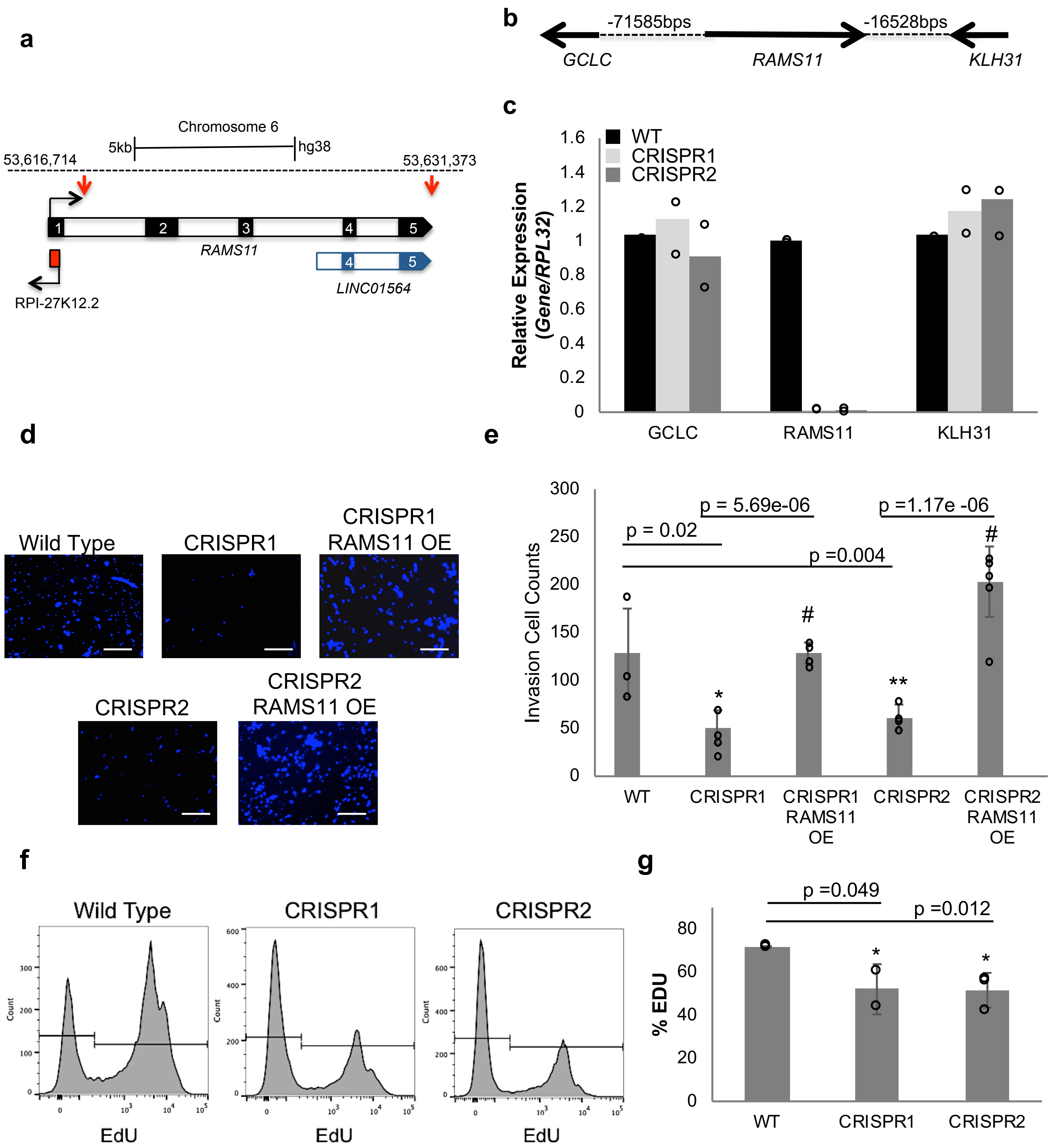

Supplementary Figure. 2: *RAMS11* CRISPR characterization. (a) Region of interest for gDNA deletion. Primers shown by red arrows. Exon 1 (red) overlapped RPI-27K12.2 and was avoided. **(b)** Schematic showing distance of nearby surrounding genes and (**c**) qPCR indicating decrease only of *RAMS11* expression and not nearby genes in CRISPR cell lines. Experiment repeated two times, n = 2 samples for all. **(d and e)** Transwell images of DAPI stained LoVo Wild Type, CRISPR1 and CRISPR2 cells show decreased invasion, and CRISPR cells with *RAMS11* overexpression plasmid (OE) increased invasion. Experiment repeated three times. WT n = 4, CRISPR1 n = 7, CRISPR1 *RAMS11* OE n = 5, CRISPR2 n = 7, CRISPR1 *RAMS11* OE n = 6, Bars = 25μM **(f and g)** Flow cytometry detecting EdU (5-ethynyl-2´-deoxyuridine) incorporation in *RAMS11* CRISPR KO cells. Experiment repeated two times with all data n =3. Data is presented as mean values ± s.d and analyzed by two-tailed paired *t*-test. *p < 0.05, **p < 0.005, #p < 0.0005

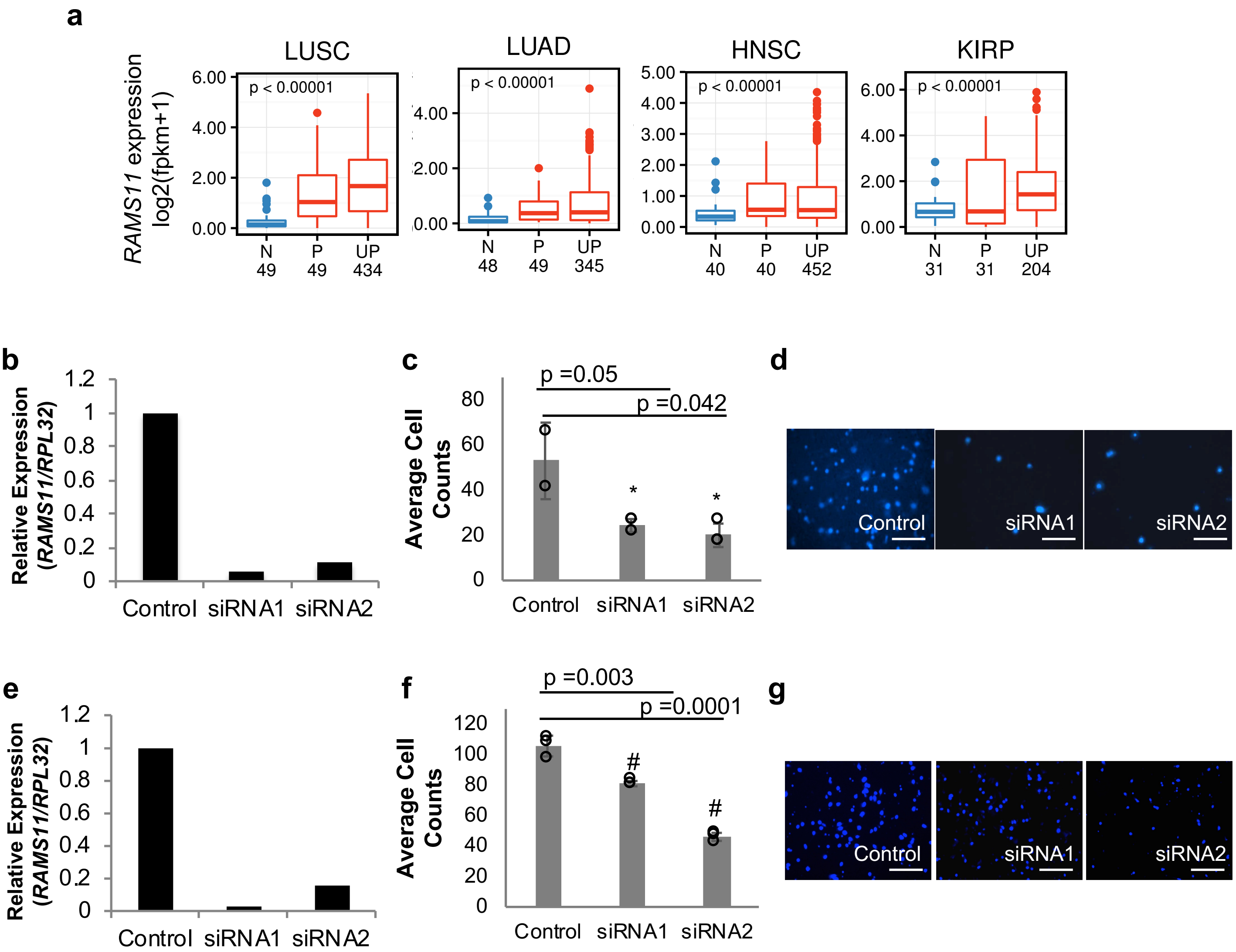

Supplementary Figure. 3: *RAMS11* is an onco-lncRNA that increases the invasive phenotype in cancer cell lines. (a) *RAMS11* up-regulation in cancer from The Cancer Genome Atlas (TCGA)**.** Shown are boxplots with box representing the interquartile range (IQR, 25th to 75th percentile) centered by median, whisker lines limited by 1.5xIQR away from the box, and dots representing outliers. HNSC pvalue = 8.76 e -06, KIRP pvalue = 9.87 e -06, LUAD pvalue = 2.12 e -09, LUSC pvalue = 1.05 e -17. Exact two-sided p-values were determined using a negative binomial model. Lung squamous cell carcinoma (LUSC), lung adenocarcinoma (LUAD), head and neck squamous cell carcinoma (HNSC), and kidney renal papillary cell carcinoma (KIRP). Normal (N), Primary, (P), and Unmatched primary (UP). **(b)** Knockdown efficiency of *RAMS11* in HCC95 LUSC cell line. **(c** and **d)** Knockdown of *RAMS11* with siRNAs shows a significant decrease in invasion in HCC95 cells. Control n = 2, siRNA1 n = 3, siRNA2 n = 3, **(e)** Knockdown efficiency of *RAMS11* in A549 LUAD cell line. **(f** and **g)** Knockdown of *RAMS11* with siRNAs shows a significant decrease in invasion in A549 cells. n = 3 DAPI stained cells are shown in blue. Data is presented as mean values ± s.d and analyzed by two-tailed paired *t*-test. Bars = 25μM. *p < 0.05, # p < 0.0005.

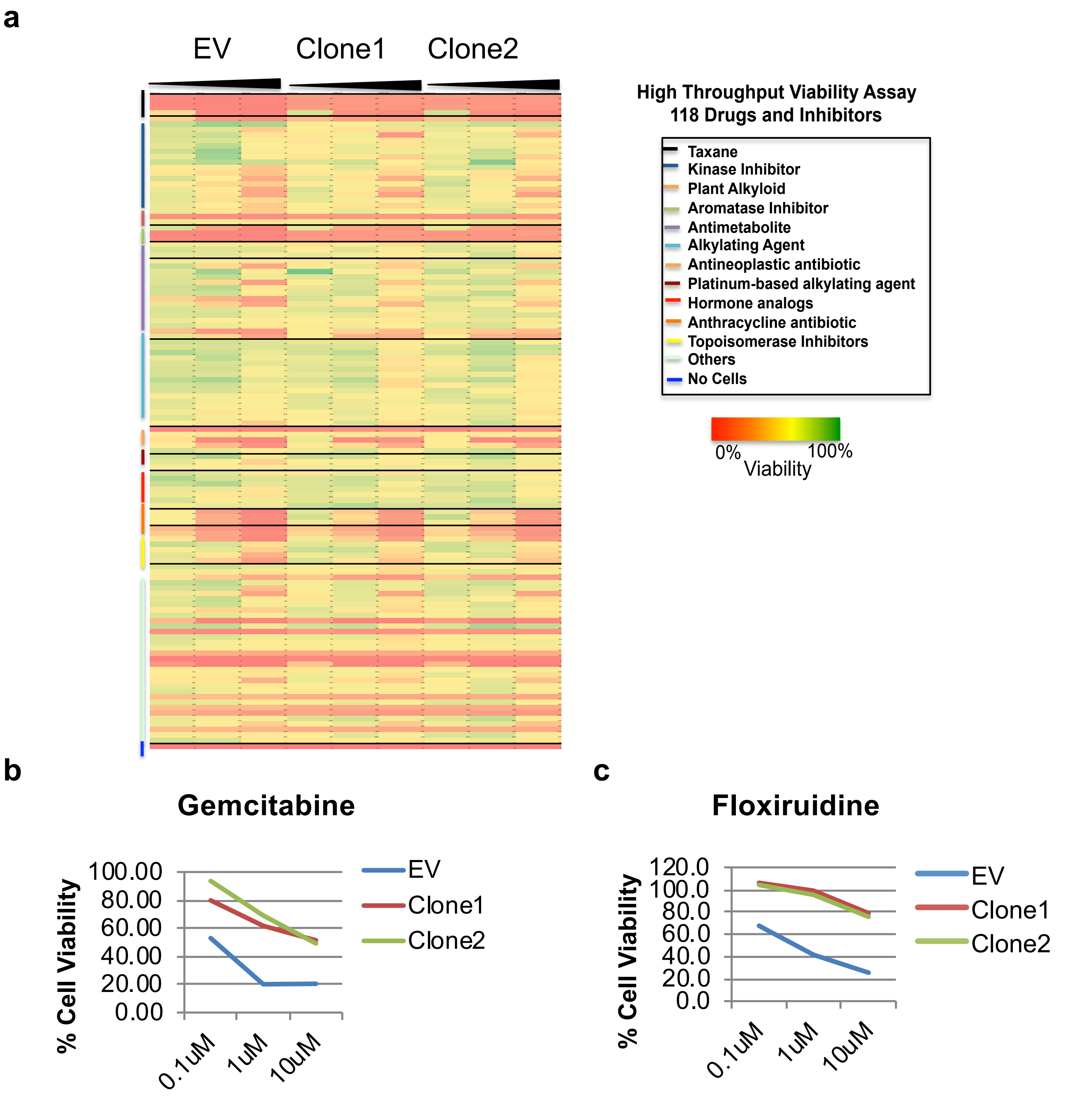

Supplementary Figure. 4: High Throughput drug viability assays reveal *RAMS11* overexpression causes drug resistance. (a) High throughput viability assay on 119 drugs with HT29 *RAMS11* overexpressing cells**.** Gemcitabine **(b)** and Floxiruidine **(c)** shows significant resistance to *RAMS11* overexpression.

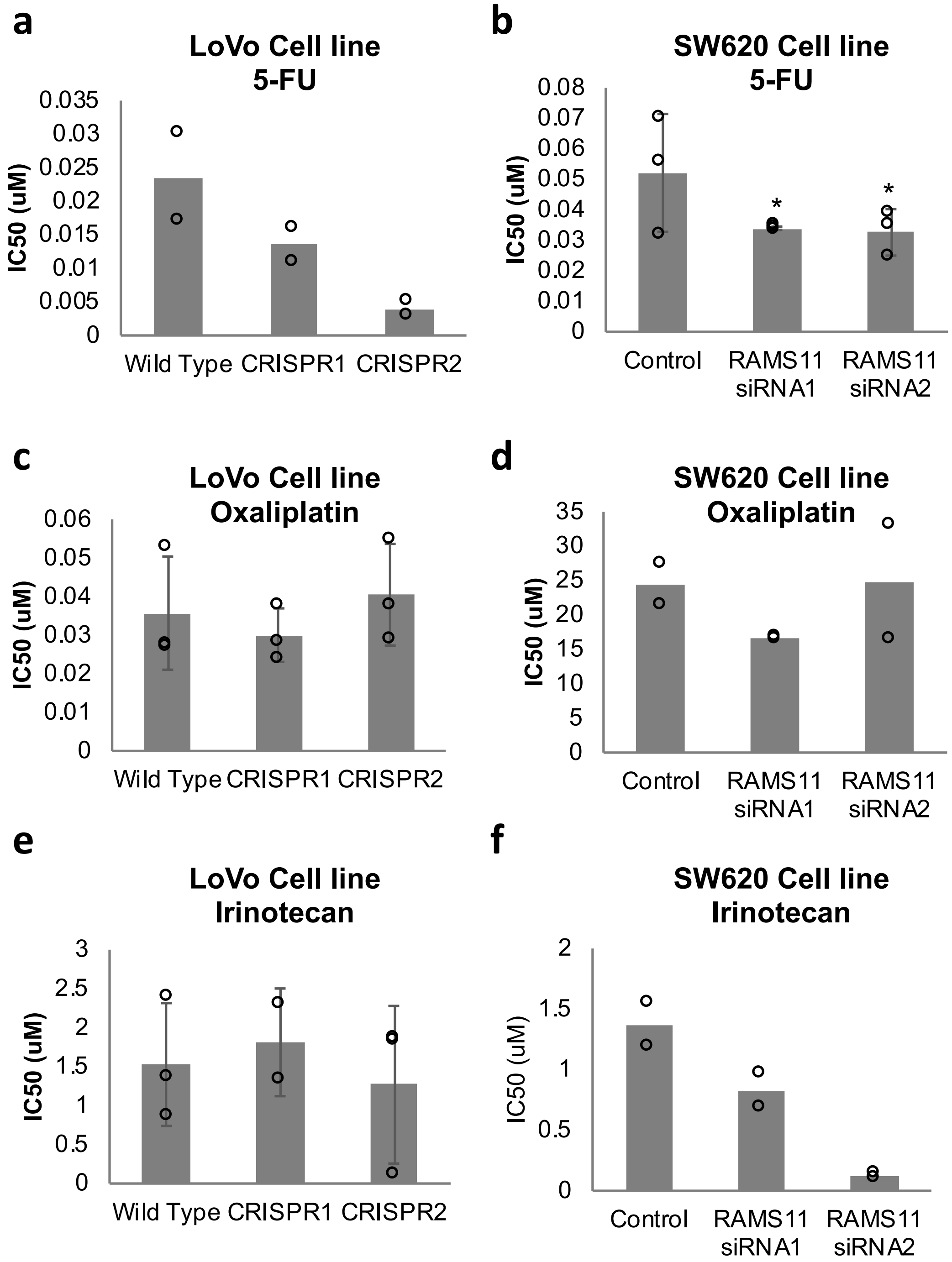

Supplementary Figure. 5: **IC50s of clinically used drugs for colorectal cancer treatment.** *RAMS11* CRISPR KO cell lines and SW620 cells with silenced *RAMS11* treated with 5-FU (**a** and **b**), Oxaliplatin **(c** and **d),** and Irinotecan **(e** and **f)** drug treatments. Data is presented as mean values ± s.d, n = 3. Experiments repeated more than two times. *Fold > 1.5, ** Fold > 5

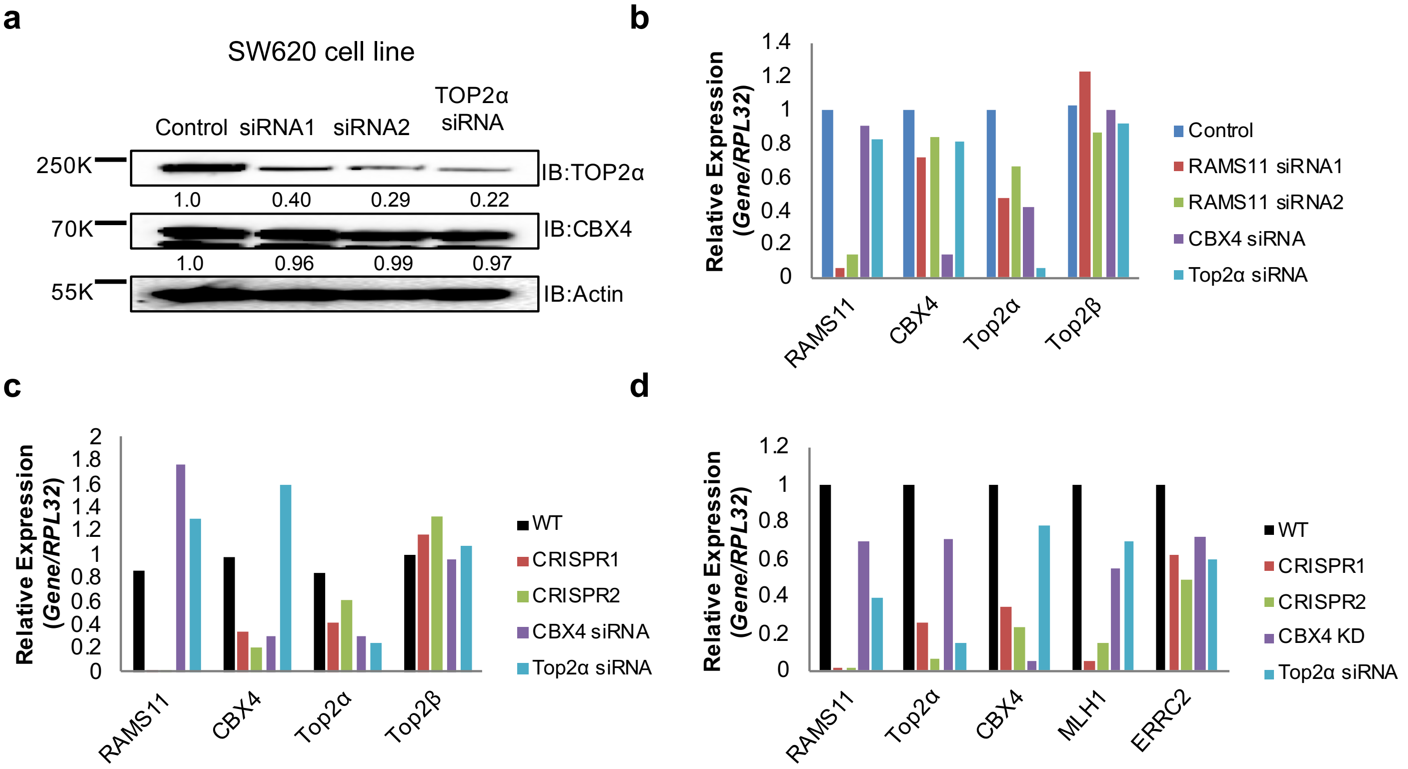

Supplementary Figure. 6: *RAMS11* regulation of Top2*α* expression. (a) Protein expression of TOP2α and CBX4 in SW620 *RAMS11* silenced cells. Band intensities were quantified from the digital image in ImageJ and are shown normalized to the Wild Type or control lane for each target. Samples derived from the same experiment and blots were processed in parallel. mRNA expression of *RAMS11, CBX4, TOP2α,* and *TOP2β* in **(b)** SW620 *RAMS11* silenced cells and **(c)** LoVo CRISPR KO cells. **(d)** Decrease in expression of *TOP2α* downstream genes with silenced *RAMS11, TOP2α,* or *CBX4.* Experiments repeated two times.

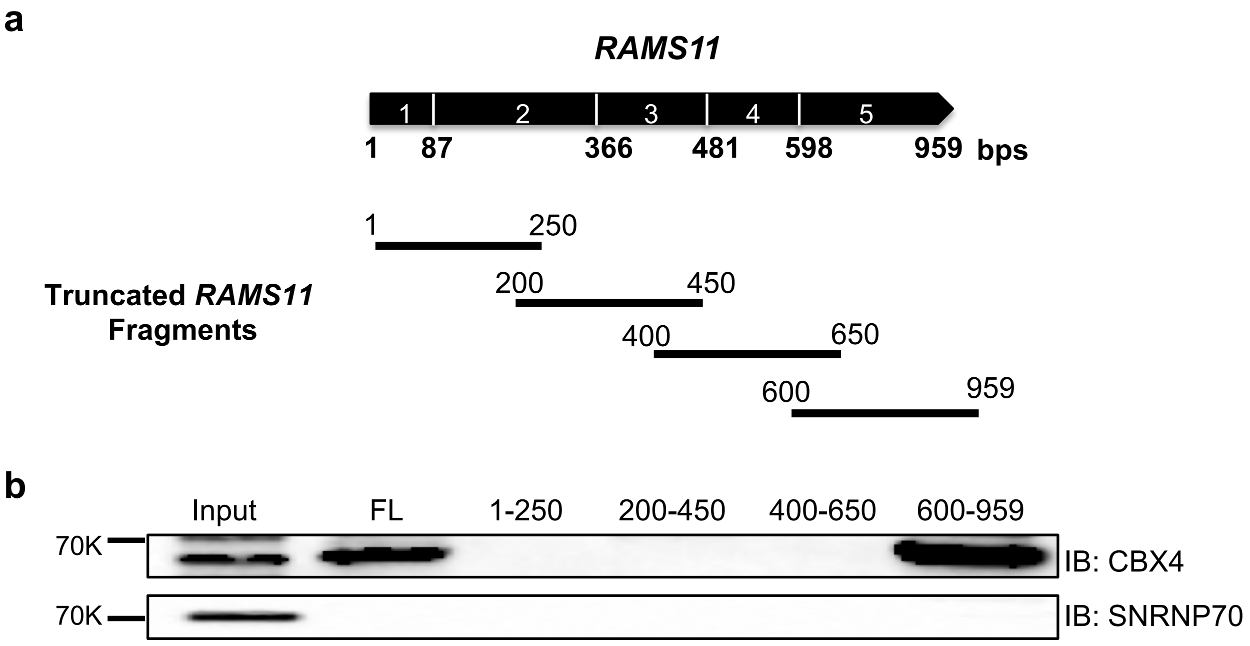

Supplementary Figure. 7: RNA pull down of truncated *RAMS11* fragments shows nucleotides 600-959 binding to CBX4. **(a)** *RAMS11* five-exon transcript (top) and four created truncated *RAMS11* fragments. **(b)** Western blot of CBX4 of RNA pull down with input, full length (FL) and four truncated *RAMS11* fragments showing interaction at 600-959 and no binding to SNRP70 negative control. Samples derived from the same experiment and blots were processed in parallel. Experiments repeated two times.

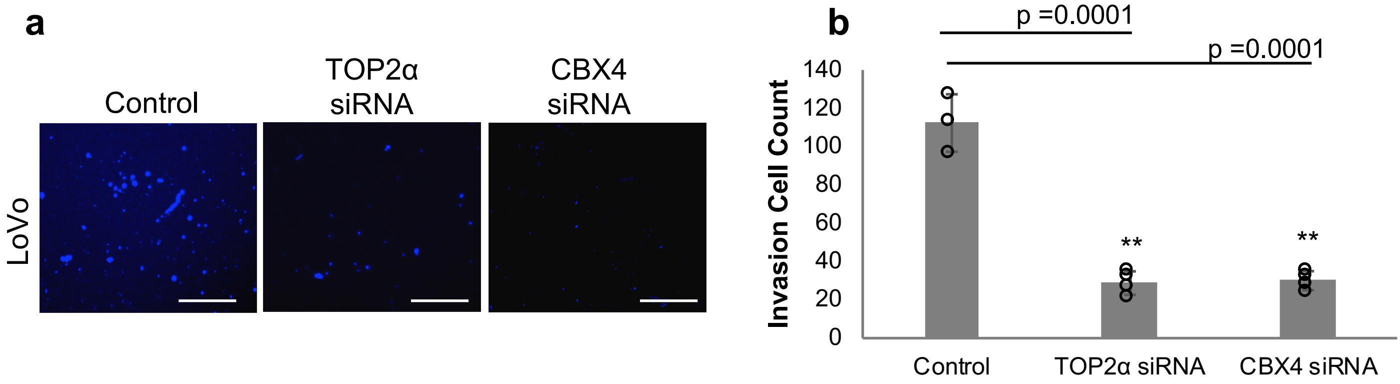

Supplementary Figure. 8: TOP2α and CBX4 promote oncogenic phenotypes. (**a**) Transwell images of invading DAPI-stained LoVo cells with silenced *TOP2α* or *CBX4.* **(b)** Quantification of transwell assay. Data is presented as mean values ± s.d, analyzed by two-tailed paired *t*-test and repeated three times. Bars = 25uM. ** $p < 0.005$.

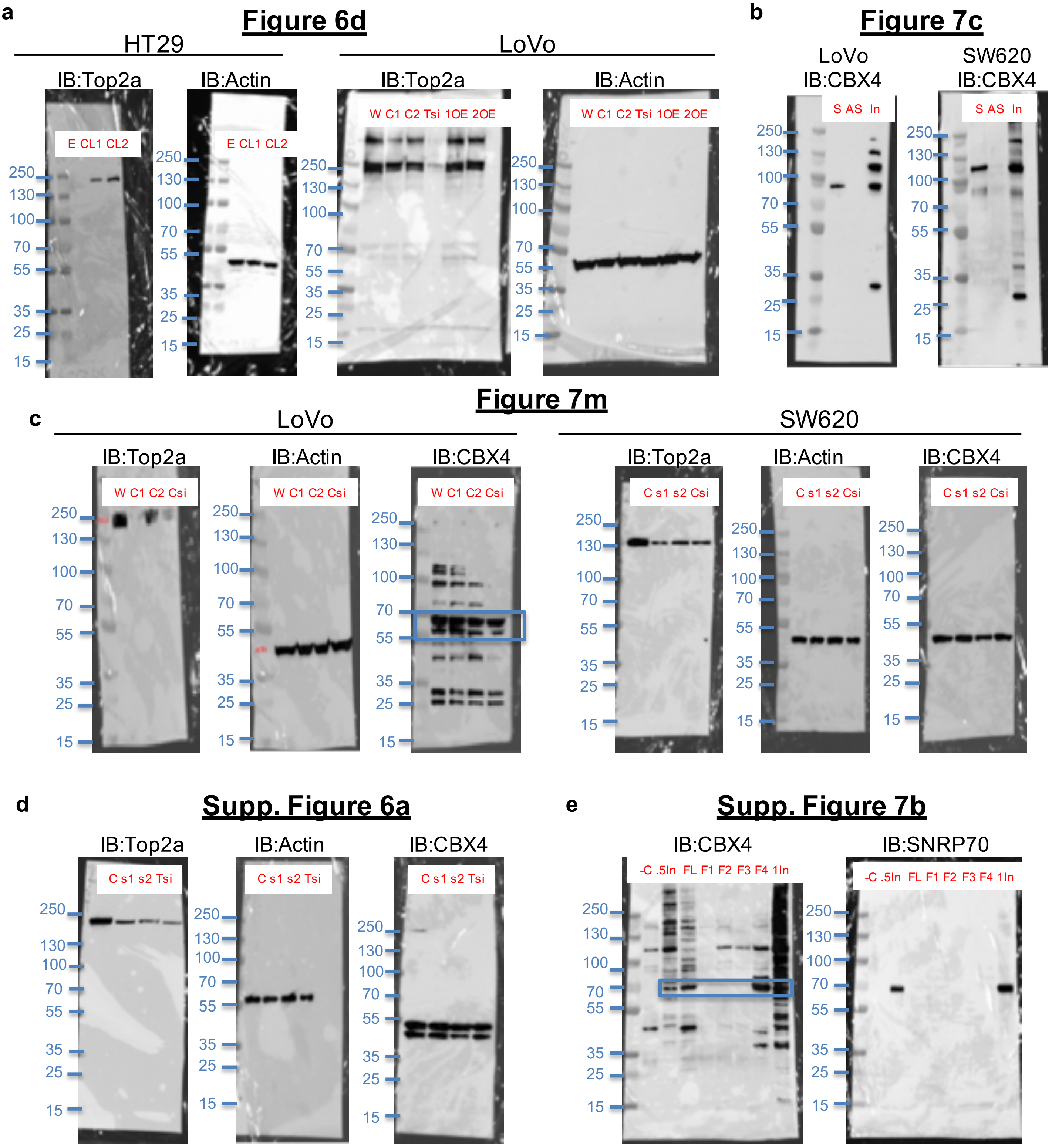

Supplementary Figure. 9: Raw blots for western blots. (a) Figure 6d western blots, (**b**) Figure 7c RNA Pull down blots, (**c**) Figure 7m blots, (**d**) Supplementary Figure 6a blots, and **(e)** Supplementary Figure 7b. Cell lines and antibodies are labeled on top of gels. Protein ladders are labeled in blue and proteins. Samples derived from the same experiment and blots were processed in parallel. E (EV), CL1 (clone1), CL2 (clone2), W (Wild Type), C1 (CRISPR1), C2 (CRISPR2), Tsi (Top2a siRNA), 1OE (CRISPR1 RAMS11 OE), 2OE (CRISPR2 RAMS11 OE), S (Sense), AS (Antisense), In (Input), Csi (CBX4 siRNA), s1 (RAMS11 sirna1), s2 (RAMS11 sirna2), -C (negative control), .5In (5% input), FL (full length), F1 (Fragment1 1-250), F2 (Fragment2 250-450), F3 (Fragment3 400-650), F4 (Fragment1 600-959), 1In (1%input)

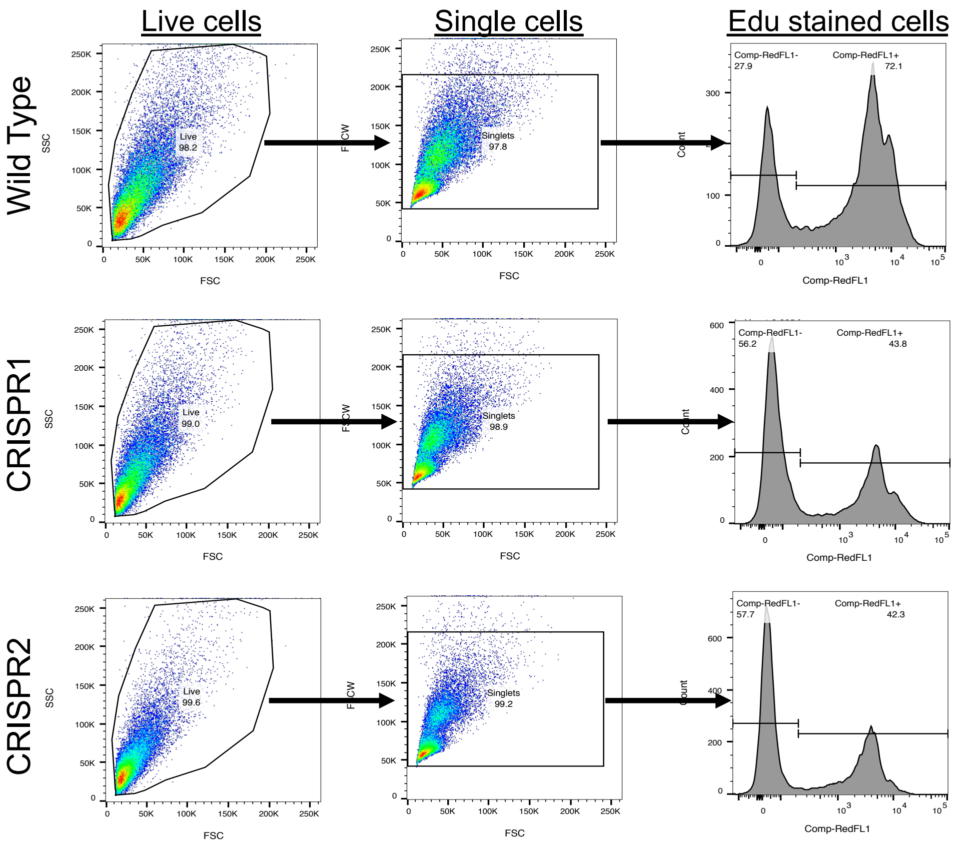

Supplementary Figure. 10: Gating strategy for Supplementary Fig 2f. LoVo Wild Type, CRISPR1, and CRISPR2 gating for live cells then single cells and Edu stained cells (RedFL+) showing decease Edu in CRISPR cell lines.
