# Supplementary material for: Long non-coding RNA RAMS11 promotes metastatic colorectal cancer progression
Source: Nat Commun. 2020 May 1;11:2156. doi: 10.1038/s41467-020-15547-8 (PMC7195452; doi:10.1038/s41467-020-15547-8)
Supplement: Supplementary file 3 — Description of Additional Supplementary Files [file 41467_2020_15547_MOESM3_ESM.docx]

**Description of Additional Supplementary Files**

**Supplementary Data 1**

List of identified differentially expressed RNAs Associated with Metastasis in colon cancer

**Supplementary Data 2**

Sequence identified for *RAMS11* 5’3’ RACE

**Supplementary Data 3**

Viability assay results from FDA approved drug panel of *RAMS11* overexpression cells

**Supplementary Data 4**

Metastatic colon cancer patient information and RNA sequencing data mapped rates

**Supplementary Data 5**

Primer lists for qPCR, CHIP, siRNAs, and RACE

**Supplementary Data 6**

List of antibodies used in manuscript
