# Supplementary material for: Long non-coding RNA RAMS11 promotes metastatic colorectal cancer progression
Source: Nat Commun. 2020 May 1;11:2156. doi: 10.1038/s41467-020-15547-8 (PMC7195452; doi:10.1038/s41467-020-15547-8)
Supplement: Supplementary file 10 — Reporting Summary [file 41467_2020_15547_MOESM10_ESM.pdf]

nature research

Corresponding author(s): Christopher Maher

Reporting Summary

Nature Research wishes to improve the reproducibility of the work that we publish. This form provides structure for consistency and transparency in reporting. For further information on Nature Research policies, see Authors & Referees and the Editorial Policy Checklist.

Statistical parameters

When statistical analyses are reported, confirm that the following items are present in the relevant location (e.g. figure legend, table legend, main text, or Methods section).

n/a	Confirmed	
☐	☒	The exact sample size (*n*) for each experimental group/condition, given as a discrete number and unit of measurement
☐	☒	An indication of whether measurements were taken from distinct samples or whether the same sample was measured repeatedly
☐	☒	The statistical test(s) used AND whether they are one- or two-sided<br>*Only common tests should be described solely by name; describe more complex techniques in the Methods section.*
☐	☒	A description of all covariates tested
☐	☒	A description of any assumptions or corrections, such as tests of normality and adjustment for multiple comparisons
☐	☒	A full description of the statistics including central tendency (e.g. means) or other basic estimates (e.g. regression coefficient) AND variation (e.g. standard deviation) or associated estimates of uncertainty (e.g. confidence intervals)
☐	☒	For null hypothesis testing, the test statistic (e.g. *F*, *t*, *r*) with confidence intervals, effect sizes, degrees of freedom and *P* value noted<br>*Give P values as exact values whenever suitable.*
☒	☐	For Bayesian analysis, information on the choice of priors and Markov chain Monte Carlo settings
☒	☐	For hierarchical and complex designs, identification of the appropriate level for tests and full reporting of outcomes
☒	☐	Estimates of effect sizes (e.g. Cohen's *d*, Pearson's *r*), indicating how they were calculated
☐	☒	Clearly defined error bars<br>*State explicitly what error bars represent (e.g. SD, SE, CI)*

Our web collection on statistics for biologists may be useful.

Software and code

Policy information about availability of computer code

Data collection	RNA sequencing of WUSTL cohort was performed using Illumina HiSeq 2000; TCGA RNA-Seq data were downloaded the cancer genomics hub and GDC data portal; Kim RNA-Seq and other exon array data were downloaded from NCBI GEO.
Data analysis	Tophat 2.0.8 was used for RNA-Seq alignment. Cufflinks 2.1.1 was used for transcript assembly; FeatureCounts v1.5.0 was used to generate read counts for transcripts; EdgeR 3.8.6 was used for differential expression analysis; SeqMap 1.0.12 was used to align exon array probes to transcript sequences; R Survival package 2.37-7 and Survplot 0.0.7 was used for survival analysis. Affymetrix Power Tool 1.18 was used to process and normalize exon array expression data. Exon array probe sequences aligned to the transcript sequences using SeqMap 1.0.12 [Jiang et al PMID: 18697769] allowing one mismatch. Only probe sets consisting of probes that were uniquely aligned to transcripts from the same genes were retained. Exon array expression was processed and normalized using Affymetrix Power Tool 1.18 (https://www.thermofisher.com). Survival analysis was performed using the Cox proportional hazard model with R survival package 2.37-7 [cite Therneau T. A Package for Survival Analysis in S. R package version 2.37-7, 2014.]. Kaplan-Meier curves were plotted using the R survplot package 0.0.7 [cite Eklund A. survplot: Plot survival curves with number-at-risk. R package version 0.0.7, 2014.].<br>Q Capture pro version 7 was used for capturing flourescent imaging and Image J version 1.15a was used for quantifying DAPI stained images. Living Image version 2.6 was used for visualizing and quantifying in vivo mouse models images.

For manuscripts utilizing custom algorithms or software that are central to the research but not yet described in published literature, software must be made available to editors/reviewers upon request. We strongly encourage code deposition in a community repository (e.g. GitHub). See the Nature Research guidelines for submitting code & software for further information.

Data

Policy information about availability of data

All manuscripts must include a data availability statement. This statement should provide the following information, where applicable:

- Accession codes, unique identifiers, or web links for publicly available datasets
- A list of figures that have associated raw data
- A description of any restrictions on data availability

The data associated with this publication are available in dbGaP, study accession phs001722.v1.p1

Field-specific reporting

Please select the best fit for your research. If you are not sure, read the appropriate sections before making your selection.

☒ Life sciences ☐ Behavioural & social sciences ☐ Ecological, evolutionary & environmental sciences

For a reference copy of the document with all sections, see nature.com/authors/policies/ReportingSummary-flat.pdf

Life sciences study design

All studies must disclose on these points even when the disclosure is negative.

Sample size	The sample size for in vivo subcutaneous and tail vein model work was 10-18 mice per group and allowed us to detect a minimum of 1.5 SD between-group differences, where SD is the standard deviation in the pooled data.
Data exclusions	Experimental assays were excluded if positive or negative controls did not validate. IC50 experiments were excluded if undetectable or out of range by resazurin assay detection and or controls did not work. Data exclusions were pre-established.
Replication	qPCR was replicated at least in triplicate to verify knock down (KD) or overexpression (OE) of representative genes. Western blots were replicated at least in triplicate. RNA immunoprecipitation and chromatin immunoprecipitation was repeated at least in duplicate. BrU-labeled RNA pulldowns were repeated in at least 2 cell lines. Nuclear cytoplasmic extractions were repeated at least in triplicate in 2-3 different cell lines. Transwell assays were repeated at least in triplicate in multiple cell lines. Soft agar assays were repeated at least in triplicate. IC50 assays for validation of specific drugs were repeated at least in triplicate. In vivo model experiments were repeated at least in duplicate. Not all replications were successful and were not included primarily due to not sufficient knockdown or incorrect nuclear cytoplasmic extraction. and were repeated.
Randomization	Randomization was not relevant to this study. We compared specific samples or cohorts to each other. We were aware of the cell lines used for each study and ensured live cell counts were the same prior to seeding in vitro or in vivo experiments.
Blinding	The collaborator who conducted and analyzed the NIH drug panel and soft agar assays were blinded by study groups. Blinding for mouse experiments was not required for this experiment as we were specifically comparing cohorts with or without lncRNA expression and was not required.

Reporting for specific materials, systems and methods

Materials & experimental systems

n/a	Involved in the study	
☐	☒	Unique biological materials
☐	☒	Antibodies
☐	☒	Eukaryotic cell lines
☒	☐	Palaeontology
☐	☒	Animals and other organisms
☐	☒	Human research participants

Methods

n/a	Involved in the study	
☒	☐	ChIP-seq
☐	☒	Flow cytometry
☒	☐	MRI-based neuroimaging

Unique biological materials

Policy information about availability of materials

Obtaining unique materials	Unique materials such as CRISPR cell lines or overexpression cell lines are available upon request from the authors.

Antibodies

Antibodies used	Top2A, Cell Signaling, 12286S, Lot:1<br>CBX4, Abcam, ab139815,Lot:GR1399769-1<br>Actin, Cell Signaling, 3700S, Lot:15<br>Anti-rabbit HRP linked, Thermo, 7074P2,<br>Goat anti-mouse HRP-linked, Thermo, 31430,<br>IgG, Cell Signaling, 2729S,<br>H3K4me3, Abcam, ab12209, Lot:GR3185135-6<br>Ki67, Cell Signaling, 9027S, Lot:6<br>SNRP70, EMD Millipore, 03-103, Lot:3211991
Validation	Cell Signaling antibodies as indicated by manufacturer are validated to verify specificity, lot-to-lot testing, and isotype controls.<br>Top2A: We verified specificity using a Top2A siRNA in this manuscript. Citations from manufacturer are listed at https://www.citeab.com/antibodies/1201490-12286-topoisomerase-ii-d10g9-xp-rabbit-mab?utm_campaign=Widget+All+Citations&utm_medium=Widget&utm_source=Cell+Signaling+Technology.<br>Actin manufacture citations are listed here: https://www.citeab.com/antibodies/123338-3700-actin-8h10d10-mouse-mab?utm_campaign=Widget+All+Citations&utm_medium=Widget&utm_source=Cell+Signaling+Technology.<br>IgG manufacture citations listed here:https://www.citeab.com/antibodies/654207-2729-normal-rabbit-igg?utm_campaign=Widget+All+Citations&utm_medium=Widget&utm_source=Cell+Signaling+Technology<br>Ki67 manufacture citations listed here:https://www.citeab.com/antibodies/654333-9027-ki-67-d2h10-rabbit-mab-ihc-specific?utm_campaign=Widget+All+Citations&utm_medium=Widget&utm_source=Cell+Signaling+Technology<br><br>Activity, stability and performance are important checks carried out by Abcam for validation of antibodies (listed on manufacture website).<br>CBX4: We use a CBX4 siRNA to verify specificity in this manuscript. Citation: Pradeepa MM et al. Psip1/Ledgf p75 restrains Hox gene expression by recruiting both trithorax and polycomb group proteins. Nucleic Acids Res 42:9021-32 (2014). ChIP .<br>H3K4me3: Manufacture citations listed here: https://www.abcam.com/histone-h3-tri-methyl-k4-antibody-mabcam12209-chip-grade-ab12209-references.html#top-369<br><br>Thermo Fisher antibodies are tested in two parts for 1) target specificity and 2) functional application.<br>IgG Mouse citations are listed here:https://www.thermofisher.com/antibody/product/Goat-anti-Mouse-IgG-H-L-Secondary-Antibody-Polyclonal/31430<br>IgG Rabbit citations are listed here:https://www.thermofisher.com/antibody/product/Goat-anti-Rabbit-IgG-H-L-Secondary-Antibody-Polyclonal/65-6120

Eukaryotic cell lines

Policy information about cell lines

Cell line source(s)	Colon cancer cell lines CCD18-Co and SW480 were a kind gift from Dr. David Shalloway at Cornell University. All other colon cell lines (HT-29, HT-15, DLD1, SW620, Caco-2, HCT-116, and RKO) were a kind gift from Dr. A. Craig Lockhart at Washington University. LoVo cell lines were purchased from ATCC. HCC95 and A549 cell lines were a kind gift from Drs. Loren Michel and Brian Van Tine at Washington University School of Medicine. All cell lines are obtained commercially.
Authentication	LoVo cell lines were authenticated by ATCC. ATCC uses morphology, karyotyping, and PCR based approaches to confirm the identity of human cell lines and to rule out both intra- and interspecies contamination. Our lab did not authenticate cells lines that were a gift.
Mycoplasma contamination	Cell lines were tested for mycoplasma contamination. If they were positive for mycoplasma we treated cell lines with Invitrogen Plasmocure (cat#ant-pc) for 2 weeks until results came back negative.
Commonly misidentified lines<br>(See ICLAC register)	No commonly misidentified cell lines are used.

Animals and other organisms

Policy information about studies involving animals; ARRIVE guidelines recommended for reporting animal research

Laboratory animals	Female, 5 weeks old, NOD/SCID mice; Female, 8 weeks old, NGS mice, Mice are housed under specific pathogen-free (SPF) barrier conditions in HEPA-filtered, individually ventilated caging (Allentown NexGen) with autoclaved 1/4 inch corncob bedding. Diet consists of irradiated PicoLab 5058 or 5053 (regular and breeder diets, respectively) with filtered 2 ppm chlorinated water ad libitum.
Wild animals	Study did not involve wild animals
Field-collected samples	Study did not involve field-collected samples

Human research participants

Policy information about studies involving human research participants

Population characteristics	Patients are primarily recruited from the multidisciplinary Siteman Cancer Center (SCC) colorectal cancer program and the clinics of Surgical and Medical Oncology. The primary service area of the SCC is the St. Louis Greater Metropolitan Area (GMA), which comprises 12 counties (seven in Missouri, five in Illinois) that include approximately 2.6 million residents. While this area is primarily made up of Caucasians, there is a large population of African Americans (approximately 18%), with smaller numbers of Asian, Hispanic, and other minorities. Colorectal cancer patients ranged in age from 41 to 71 and included both male and female patients.
Recruitment	Patients were recruited from the colorectal cancer clinics at Washington University. The consent process required that they be informed by a physician that their participation is not intended to benefit them directly but to help future patients with cancer and that they may withdraw consent at any time. All patients with biopsy-proven adenocarcinoma of the colon and rectum were eligible for participation. All pertinent demographic, patient, pre-treatment, treatment, and follow-up information was collected and tracked prospectively as metadata for subsequent analysis. There was no inclusion or exclusion criteria for recruitment thus has no potential self-selection bias.

Flow Cytometry

Plots

Confirm that:

- [x] The axis labels state the marker and fluorochrome used (e.g. CD4-FITC).
- [x] The axis scales are clearly visible. Include numbers along axes only for bottom left plot of group (a 'group' is an analysis of identical markers).
- [x] All plots are contour plots with outliers or pseudocolor plots.
- [x] A numerical value for number of cells or percentage (with statistics) is provided.

Methodology

Sample preparation	LoVo colon cancer CRISPR cell lines were used for flow cytometry.
Instrument	Becton Dickinson FACScan
Software	FlowJo software v10 was used for data collection and analysis.
Cell population abundance	25,000 cells were assessed and greater than 97% live single cells were used for each cell population.
Gating strategy	Greater than 97% live cells FSC/SSC gates were used for each starting cell population. We next used single cells to determine positive cells stained with RedFL+ showing Edu stain. This is highlighted in Supplementary Figure 10.

- [x] Tick this box to confirm that a figure exemplifying the gating strategy is provided in the Supplementary Information.
